# FAP imaging in rare cancer entities—first clinical experience in a broad spectrum of malignancies

**DOI:** 10.1007/s00259-021-05488-9

**Published:** 2021-08-03

**Authors:** K. Dendl, R. Finck, F. L. Giesel, C. Kratochwil, T. Lindner, W. Mier, J. Cardinale, C. Kesch, M. Röhrich, H. Rathke, H. Gampp, J. Ristau, S. Adeberg, D. Jäger, J. Debus, U. Haberkorn, S. A. Koerber

**Affiliations:** 1 Department of Nuclear Medicine, Heidelberg University Hospital, Heidelberg, Germany; 2 National Center for Tumor Diseases (NCT), Heidelberg, Germany; 3 Department of Nuclear Medicine, Düsseldorf University Hospital, Düsseldorf, Germany; 4 Department of Urology, German Cancer Consortium (DKTK), University Hospital Essen, University of Duisburg-Essen, Essen, Germany; 5 Department of Cardiology, University of Heidelberg, Heidelberg, Germany; 6 Department of Radiation Oncology, Heidelberg University Hospital, INF 400, 69120 Heidelberg, Germany; 7 Heidelberg Institute of Radiation Oncology (HIRO), Heidelberg, Germany; 8 Heidelberg Ion-Beam Therapy Center (HIT), Department of Radiation Oncology, Heidelberg University Hospital, Heidelberg, Germany; 9 German Cancer Consortium (DKTK), partner site Heidelberg, Heidelberg, Germany; 10 Clinical Cooperation Unit Radiation Oncology, German Cancer Research Center (DKFZ), Heidelberg, Germany; 11 Clinical Cooperation Unit Nuclear Medicine, German Cancer Research Center (DKFZ), Heidelberg, Germany; 12 Translational Lung Research Center Heidelberg, Member of the German Center for Lung Research DZL, Heidelberg, Germany

**Keywords:** FAP imaging, ^68^ Ga-FAPI-PET/CT scan, ^68^ Ga-FAPI

## Abstract

**Purpose:**

^68^ Ga-FAPI (fibroblast activation protein inhibitor) is a rapidly evolving and highly promising radiotracer for PET/CT imaging, presenting excellent results in a variety of tumor entities, particularly in epithelial carcinomas. This retrospective analysis sought to evaluate the potential and impact of FAPI-PET/CT in rare cancer diseases with respect to improvement in staging and therapy, based on tracer uptake in normal organs and tumors.

**Material and methods:**

Fifty-five patients with rare tumor entities, defined by a prevalence of 1 person out of 2000 or less, received a ^68^ Ga-FAPI-PET/CT scan. Fourteen women and 41 men (median age 60) were included within the following subgroups: cancer of unknown primary (*n* = 10), head and neck cancer (*n* = 13), gastrointestinal and biliary-pancreatic cancer (*n* = 17), urinary tract cancer (*n* = 4), neuroendocrine cancer (*n* = 4), and others (*n* = 7). Tracer uptake was quantified by standardized uptake values SUVmax and SUVmean and the tumor-to-background ratio (TBR) was determined (SUVmax tumor/SUVmean organ).

**Results:**

In 20 out of 55 patients, the primary tumor was identified and 31 patients presented metastases (*n* = 88), characterized by a high mean SUVmax in primary (10.1) and metastatic lesions (7.6). The highest uptake was observed in liver metastases (*n* = 6) with a mean SUVmax of 9.8 and a high TBR of 8.7, closely followed by peritoneal carcinomatosis (*n* = 16) presenting a mean SUVmax of 9.8 and an excellent TBR of 29.6. In terms of the included subgroups, the highest uptake regarding mean SUVmax was determined in gastrointestinal and biliary-pancreatic cancer with 9.8 followed closely by urinary tract cancer with 9.5 and head and neck cancer (9.1).

**Conclusion:**

Due to excellent tumor visualization and, thereby, sharp contrasts in terms of high TBRs in primary and metastatic lesions in different rare malignancies, ^68^ Ga-FAPI-PET/CT crystallizes as a powerful and valuable imaging tool, particularly with respect to epithelial carcinomas, and therefore an enhancement to standard diagnostics imaging methodologies. The realization of further and prospective studies is of large importance to confirm the potential of FAP imaging in oncology.

**Supplementary Information:**

The online version contains supplementary material available at 10.1007/s00259-021-05488-9.

## Introduction

At present, no universal definition of rare tumor entities exists. In the European Union (EU), a rare disease is defined by a prevalence of no more than 1 person out of 2000. This indicates that approximately 7000 rare diseases have an impact on about 30 million people in the EU, highlighting the importance of a reliable and accurate staging tool despite low incidences [1]. A novel nuclear tracer, fibroblast activation protein inhibitor (FAPI), presented highly promising results in previously published analyses for imaging different types of cancer [2–4]. FAP, a cell membrane-bound type II serine protease, is overexpressed by cancer-associated fibroblasts (CAFs) which are associated with cancer-promoting processes and poor prognosis [5, 6]. Furthermore, several tumor entities demonstrated strong expressions of FAP, such as 90% of all epithelial carcinomas [7], enabling precise and accurate diagnostic staging and, therefore the most suitable therapeutic approach for each individual patient. Rare tumors are of particular interest as most of them originate from epithelial tissue, particularly in head and neck and biliary tract cancers [8, 9]. Additionally, FAPI might be useful for detecting peritoneal carcinomatosis, facilitating the diagnosis and guiding treatment which is rather challenging for tracers like ^18^F-FDG due to high bowel activity and therefore extensive background accumulation [10, 11]. Thus, it is of essence to evaluate the potential of ^68^ Ga-FAPI-positron emission tomography/computed tomography (PET/CT) in rare diseases in order to utilize it most efficiently.

## Material and methods

### Patient cohort

We retrospectively analyzed data of 55 patients with rare oncological malignancies, defined by prevalence of 1 person out of 2000 or less. All patients were referred by their treating oncologist in order to supplement or complete standard imaging. Indications for performing FAP imaging include insufficient delineation, inconclusive findings, or the need to select target-positive patients for last-line experimental FAP-radioligand therapy. Six out of these 55 patients were already investigated under a different scientific scope (25, 26, Röhrich and Syed et al. currently under review). All patients gave written informed consent to undergo ^68^ Ga-FAPI-PET/CT on an individual patient basis following national regulations and the Declaration of Helsinki. The radiopharmaceutical was applied according to the German Pharmaceutical Act §13(2b). The retrospective analysis was approved by the ethics committee of Heidelberg University (S016/2018).

### *Radiopharmaceuticals and *^*68*^* Ga-FAPI-PET/CT imaging*

A Biograph mCT Flow PET/CT scanner (Siemens Medical Solutions) was used as the PET system, following previously published protocols [2, 12]. In summary, scans were obtained with a low-dose whole-body CT without contrast media and in 3D mode (matrix, 200 × 200) and subsequently corrected for randoms, scatter, and decay. Patients were requested to self-report any new symptoms or abnormalities up to 30 min after the end of the examination. Imaging data was acquired 60 min after tracer administration. Different ^68^ Ga-labeled FAP ligands were used in our 55 patients with different rare oncological diseases: FAPI-04: *n* = 22; FAPI-46: *n* = 20; FAPI-74: *n* = 13. A median activity of 252 MBq (range 118–340) was injected. Chemical synthesis and radiolabeling was performed as described previously [3, 13–15] for all ^68^ Ga-labeled FAP ligands.

### Image evaluation

Tracer uptake in all patients was determined by quantification of mean and maximum standardized uptake values (SUVmean and SUVmax). Regarding SUV calculation, circular volumes of interest were drawn around tumor lesions on transaxial slices and automatically adapted to a 3-dimensional VOI with e.soft software (Siemens) at a 60% isocontour. Evaluation of normal organs was conducted with a 1 cm diameter (for the small organs thyroid, parotid gland, myocardium, oral mucosa, spinal cord, ovaries) or 2 cm diameter (brain, muscle, liver, pancreas, spleen, kidney, fat, aortic lumen content, lung, mamma, endometrium) sphere placed inside the organ parenchyma. Tumor-to-background ratios (TBRs) were determined for quantification of image contrast. The geometric mean of the quotients of lesion (SUVmax) to background tissue (SUVmean) formed the TBR, measured for metastases in lymph nodes (relative to fat tissue), bone (relative to bone spongiosa), liver (relative to liver parenchyma), peritoneal carcinomatosis (relative to fat), and lung (relative to lung parenchyma). Additionally, the TBRs of all identified tumors were formed in relation to blood pool, muscle, and fat tissue. The ^68^ Ga-FAPI-PET/CT scans were analyzed by one board-certified radiologist, one board-certified radiation oncologist, and two board-certified nuclear medicine physicians in consensus.

### Statistics

Evaluation of descriptive analyses of all patients included demographic and tumor-specific characteristics. For determination of SUVs, mean, median, and range were utilized. The Kruskal–Wallis test was used for comparison of different lesion types. The correlation of FAPI uptake within or outside the tumor was determined by using the geometrical mean. The correlation of FAPI uptake with epithelial and non-epithelial origin was determined using a 2-sided *t* test, and a *p*-value of < 0.05 was defined as statistically significant. All statistical analyses were performed using SPSS Statistics Version 24 (IBM, Armonk, NY, USA) and Excel for Mac Version 15.41 (Microsoft, Redmond, Washington, USA).

## Results

### Study population

Fourteen women and 41 men with a median age of 60 years were included in this study and underwent ^68^ Ga-FAPI-PET/CT. In 20 out of 55 patients, the primary tumor was identified, and 31 patients had metastatic lesions (*n* = 88). The following rare oncological entities were enclosed: cancer of unknown primary (*n* = 10), head and neck cancer (*n* = 13), gastrointestinal and biliary-pancreatic cancer (*n* = 17), urinary tract cancer (*n* = 4), and neuroendocrine cancer (*n* = 4). The pooled group of others contained follicular lymphoma (*n* = 1), skin cancer (*n* = 2), epithelial hemangioendothelioma (*n* = 1), multiple myeloma (*n* = 1), and oncogenic osteomalacia (*n* = 1) (supplement Table 1).

### Biodistribution and uptake in tumor lesions

**Fig. 1:**
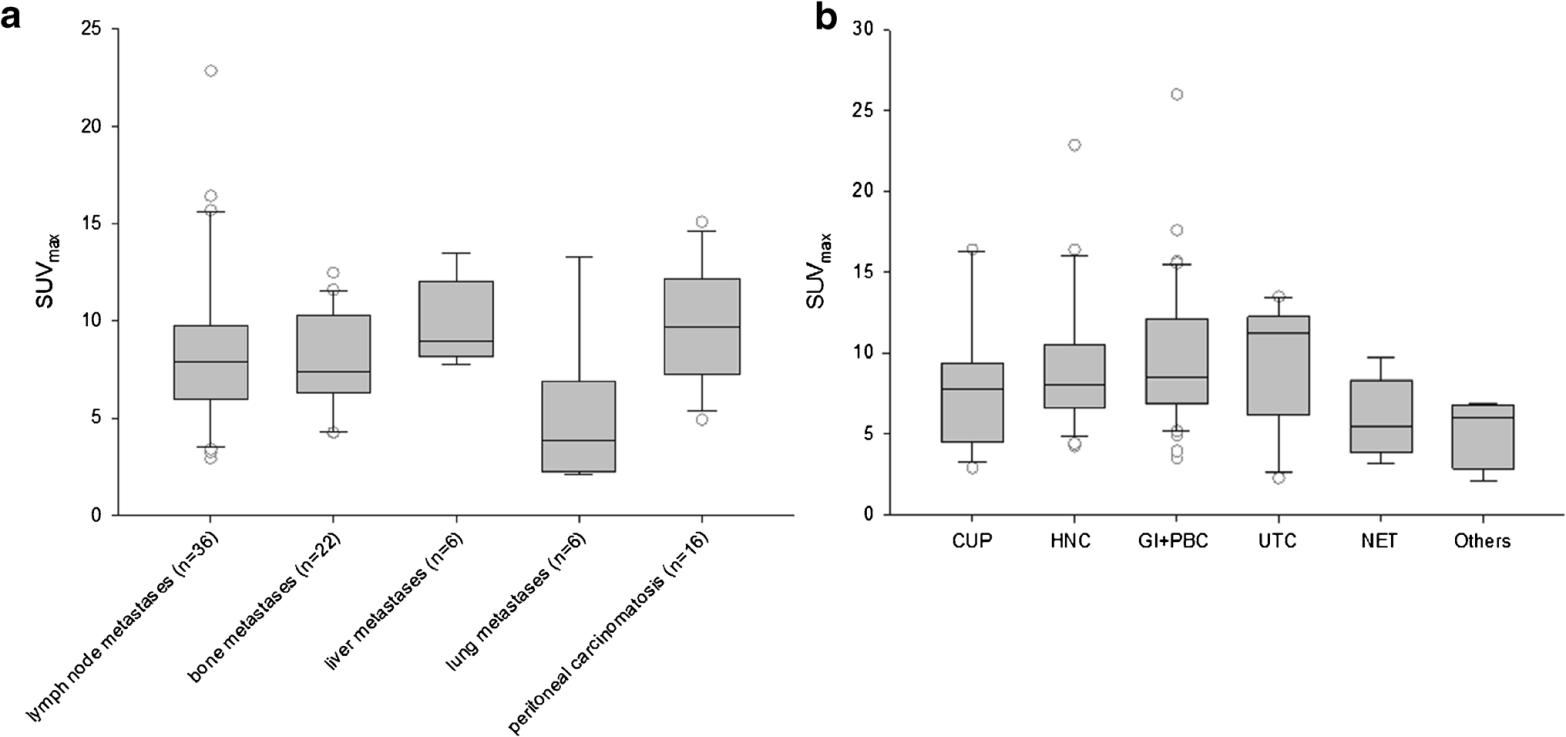
**a** Illustration of SUVmax imaged at 1 h p.i. with regard to all metastatic lesions. **b** Illustration of the different included subtypes: Cancer of unknown primary (CUP, *n* = 10), head and neck cancer (HNC, *n* = 13), gastrointestinal + pancreaticobiliary cancer (GI + PBC, *n* = 17), urinary tract cancer (UTC, *n* = 4), neuroendocrine tumor (NET, *n* = 4), others (*n* = 7)

Biodistribution of ^68^ Ga-FAPI demonstrated low background activity in most normal organs with a mean uptake of 0.9 (SUVmean) and 1.3 (SUVmax). Twenty out of 55 patients presented primary tumor lesions, and in 31 patients, 88 metastases were measured. Primary lesions consisted of primary tumors (*n* = 13) and local relapses (*n* = 7) showing a mean SUVmax of 10.1 (range 2.8–26.0) and a SUVmean of 5.5 (range 1.8–14.9). All metastases (*n* = 88) presented a mean SUVmax of 7.6 (range 2.3–13.5) and a SUVmean of 4.4 (range 1.8–7.9). The highest uptake was obtained by liver metastases (*n* = 6) and equally by peritoneal carcinomatosis (*n* = 16) with a mean SUVmax of 9.8. Strong uptakes were also determined in lymph node metastases (*n* = 36) with a mean SUVmax of 8.7, bone metastases (*n* = 22) showing a mean SUVmax of 7.8, lung metastases (*n* = 6) with a mean SUVmax of 5.0, and other metastases (*n* = 2) with a mean SUVmax of 5.2 (Fig. 1a). The highest uptake was determined in gastrointestinal and biliary-pancreatic cancer (*n* = 17) with a mean SUVmax of 9.8, followed closely by urinary tract cancer (*n* = 4) with a mean SUVmax of 9.5. Rather high uptake was also obtained in head and neck cancer (mean SUVmax 9.1), cancers of unknown primary (mean SUVmax of 7.7), and neuroendocrine tumors (mean SUVmax of 6.0). The other entities, encompassing all remaining rare diseases, presented a mean SUVmax of 5.2 (Fig. 1b). The subgroup analysis regarding cholangiocellular carcinoma (CCC) (*n* = 5) and gastric cancer (*n* = 5) established remarkably high SUV metrics. CCC presented a strong mean SUVmax of 14.7 in primary (*n* = 4) and a mean SUVmax of 10.03 in secondary lesions (*n* = 16). Gastric cancer demonstrated rather high mean SUVmax values in the primary tumor (6.8; *n* = 2) and metastases (10.23; *n* = 10) as well. By comparing different lesion including all malignancies, we determined a mean SUVmax of 10.1 in primary lesions (*n* = 20), 8.7 in lymph node metastases (*n* = 36), 9.8 in peritoneal carcinomatosis (*n* = 16), and 7.6 in distant metastases (*n* = 36). However, no significant differences in terms of uptake between those subgroups were established (*p* = 0.177). Primary and secondary lesions of epithelial origin were characterized by a higher uptake than non-epithelial lesions, as indicated by a mean SUVmax of 9.1 (*n* = 42) compared to 7.9 (*n* = 44) (*p* = 0.178), respectively.

### Tumor to background ratios

Primary tumor ratios (*n* = 20) compared to background activity of all patients presented sharp contrasts in terms of high geometrical means (tumor/fat 25; tumor/muscle 11.2; tumor/blood pool 6.8). Furthermore, excellent tumor to background ratios of 28 in lymph node metastases to fatty tissue (*n* = 36), 8.2 in bone metastases to bone tissue (*n* = 22), 3.8 in lung metastases to lung tissue (*n* = 6), 8.7 in liver metastases to liver tissue (*n* = 6), and 29.6 in peritoneal carcinomatosis to fat tissue (*n* = 16) were determined (Tables 1 and 2).

**Table 1:** TBRs of all primary lesions with regard to background uptake such as blood pool, fat tissue, and muscle tissue

	Tumor/blood pool	Tumor/fat	Tumor/muscle
TBR	6.83	24.96	11.22

**Table 2:** TBRs of all secondary lesions subdivided into lymph node, bone, liver, lung metastases, and peritoneal carcinomatosis in relation to the corresponding background tissues

	Lymph node metastases	Bone metastases	Liver metastases	Lung metastases	Peritoneal metastases
TBR	27.98	8.19	8.74	3.78	29.57

## Discussion

**Fig. 2:**
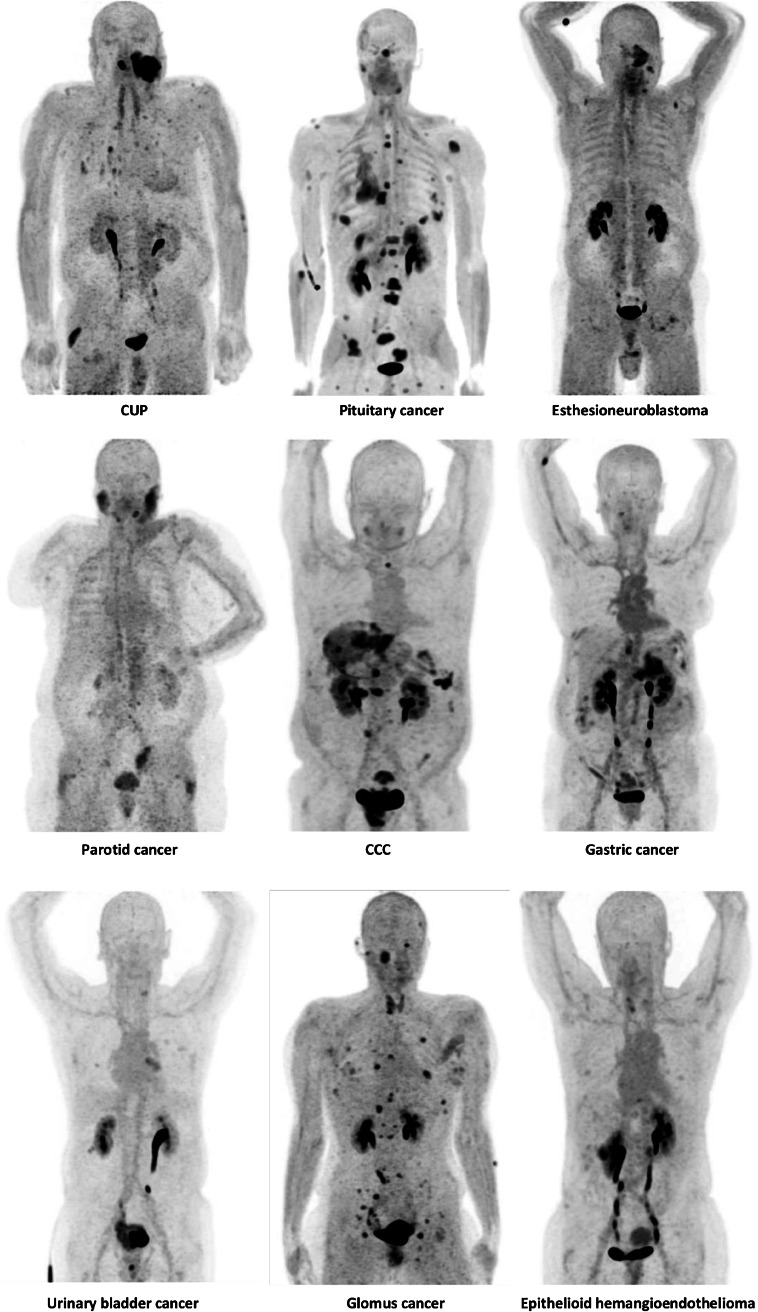
Maximum-intensity projections of ^68^ Ga-FAPI-PET/CT reflecting 9 different rare malignancies

^68^ Ga-FAPI-PET/CT proved to supplement standard diagnostics successfully in several tumor entities such as in pancreaticobiliary and gastrointestinal carcinomas, which also presented the highest uptake of all included subtypes in our analysis. A previously conducted study supports these findings, as it indicates that FAPI scans of patients with hepatocellular carcinoma (HCC) and cholangiocarcinoma (CCC) seem to be equivalent to contrast-enhanced computed tomography (CE-CT) and magnetic resonance imaging (MRI) and even superior to FDG-PET/CT [16]. Moreover, FAPI-PET/CT imaging of liver lesions was capable of successfully distinguishing between HCC, non-HCC, and normal liver parenchyma [17]. CCC (*n* = 5) in particular presented an exceedingly high mean uptake with a SUVmax of 11 (range 3.9–26). A possible explanation might be the underlying microenvironment, as CCCs are characterized by a highly desmoplastic reaction, in which cancer-associated fibroblasts are among the predominant cell types, possibly contributing to growth and therapeutic resistance [18, 19]. Simultaneously, CAFs may represent a therapeutic target [20].

This single-center retrospective analysis sought to evaluate the usefulness and impact of ^68^ Ga-FAPI-PET/CT in a cohort of patients with a variety of rare cancer diseases (Fig. 2). These are characterized by a common epithelial origin as well as low incidences and are correspondingly frequently neglected as less people are affected. Nevertheless, patients with rare malignancies should yet receive the best possible diagnostic and therapeutic approach, highlighting the immense importance of studies, particularly concerning these tumor entities.

**Fig. 3:**
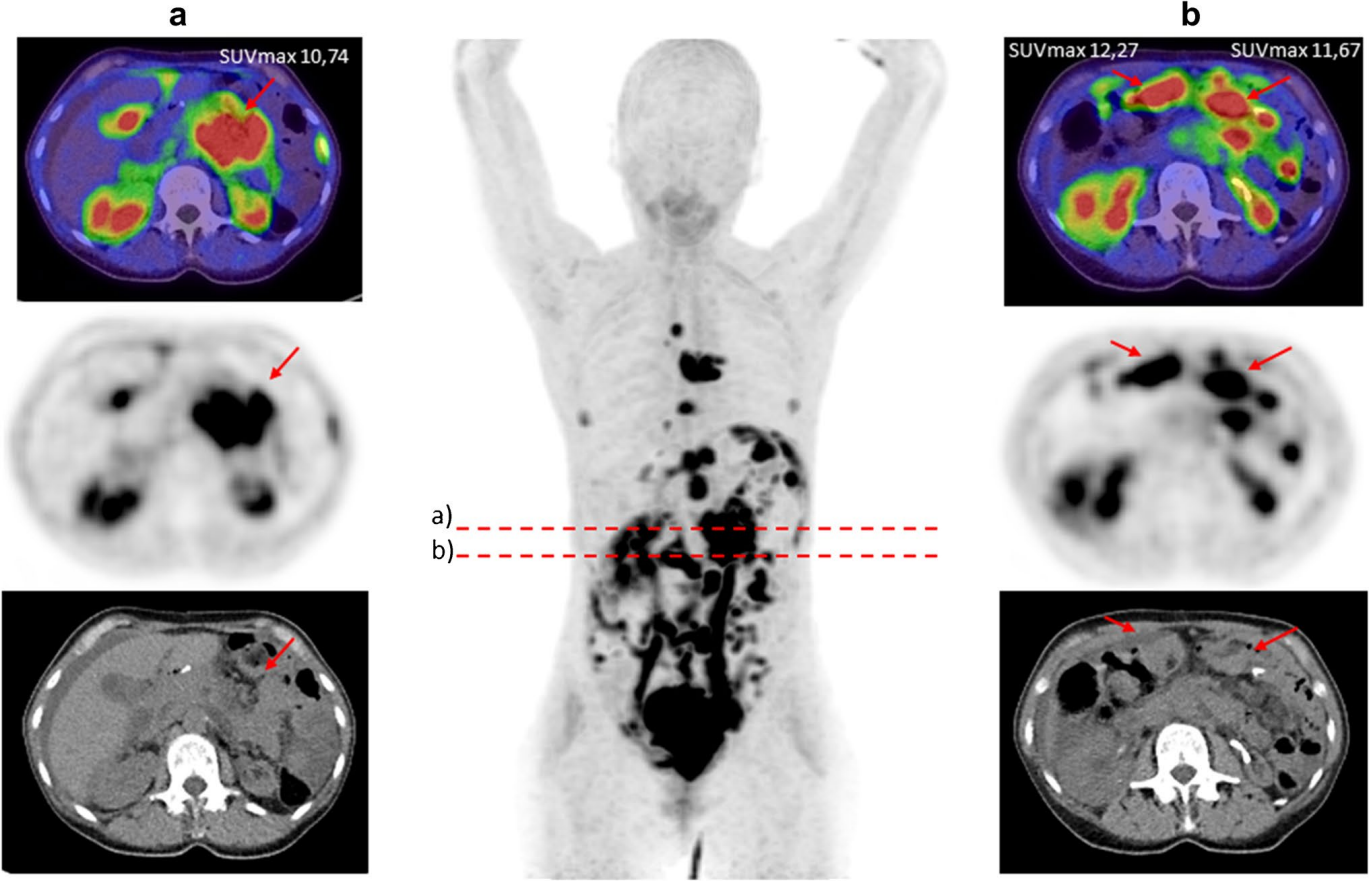
A 60-year-old female patient with gastric cancer and peritoneal carcinomatosis and lymph node metastases underwent ^68^ Ga-FAPI-PET/CT due to staging after gastrectomy. Tracer uptake of the peritoneal carcinomatosis presented an excellent median SUVmax of 11.91

^68^ Ga-FAPI-PET/CT in patients with urinary tract cancer presented a rather strong mean uptake (SUVmax 9.5) as well. For staging purposes, the European Association of Urology guidelines currently recommend contrast-enhanced CT of the chest and abdomen/pelvis [21, 22]. However, conventional CT has some limitations due to imprecise determination of the tumor allocation, extent, and invasion. As ^68^ Ga-FAPI-PET/CT most clearly demonstrated excellent tumor visualization, it may be able to complement standard diagnostic imaging and improve the precision of tumor detection, delineation, and invasion status. Furthermore, one exemplary patient posed to present equally strong uptake in the tumorous lesion only 10 min after tracer administration compared with 1 h, proving the feasibility of FAPI-PET/CT for rapid and adequate images, which may enable reduced radiation burden and improved practical implementation [3, 13–15] (Fig. 4). This patient suffered from neuroendocrine urothelial carcinoma and the favorable strong uptake demonstrates the potential of FAPI in neuroendocrine tumors, which are knowingly associated with the development of fibrosis [23, 24] and, therefore, presenting an auspicious target which has been reported in different case reports [25, 26].

Another aspect that has to be considered is the potential of FAPI-PET/CT in primarily detecting peritoneal carcinomatosis and, moreover, in improving the precision of specifying its extent. Furthermore, peritoneal carcinomatosis impressed with not only strong tracer accumulation but also excellent TBRs, allowing an improved tumor delineation. Zhao et al. evaluated the potential of FAPI-PET/CT in patients with peritoneal carcinomatosis in comparison to FDG-PET/CT, showing a clear superior peritoneal cancer index score and a higher sensitivity for FAPI in cancer patients [10]. This may be due to low physiological accumulation of ^68^ Ga-FAPI compared with ^18^F-FDG in the intestinal tract, leading to scarce unspecific uptake in the peritoneal cavity. Exceptionally favorable results regarding peritoneal carcinomatosis were obtained in patients with gastric cancer, which is in concordance with our analyses as the mean SUVmax (10.1) of patients with gastric cancer is stronger than the mean SUVmax (8.5) of all measured peritoneal carcinomatoses, enabling particularly clear imaging contrasts (Fig. 3). This may facilitate therapeutic decisions and is of assistance for radiotherapy planning with regard to target volume definition. Within the remaining tumor entities, peritoneal carcinomatosis still presented a rather high uptake, yielding the possibility to enhance standard diagnostic procedure and reduce the amount of missed diagnoses [10].

**Fig. 4:**
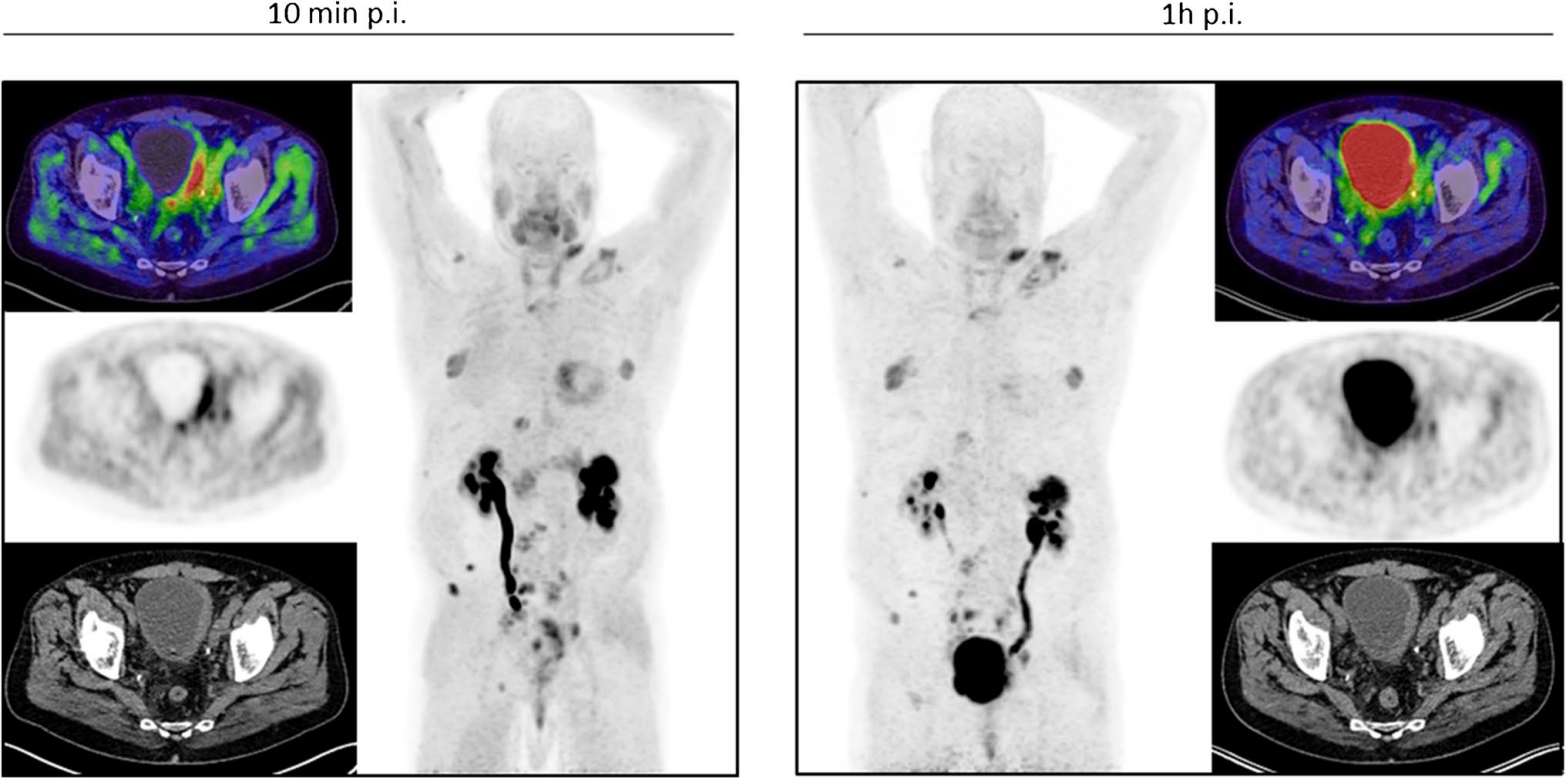
A 55-year-old male patient with a neuroendocrine metastasized urothelial bladder cancer showing similarly strong uptake 10 min (SUVmax of 9.32) and 1 h (SUVmax 9.39) after tracer administration

**Fig. 5:**
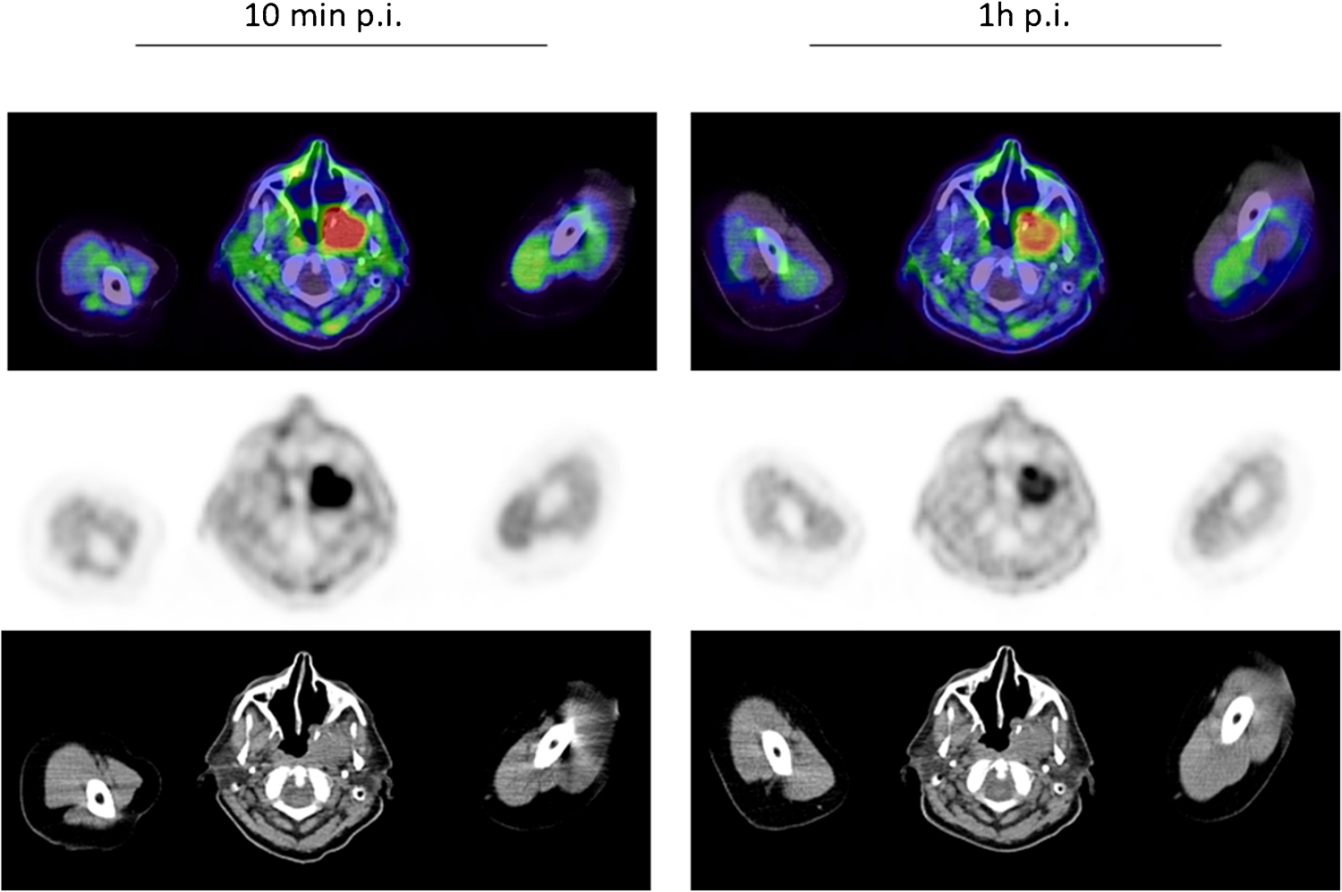
A 77-year-old patient with a retro maxillary located metastasized esthesioneuroblastoma underwent ^68^ Ga-FAPI-PET/CT due to restaging. Remarkably, the quantified tracer uptake was higher 10 min after tracer application with a SUVmax of 10.75 than 1 h p.i. with a SUVmax of 7.69, respectively

**Fig. 6:**
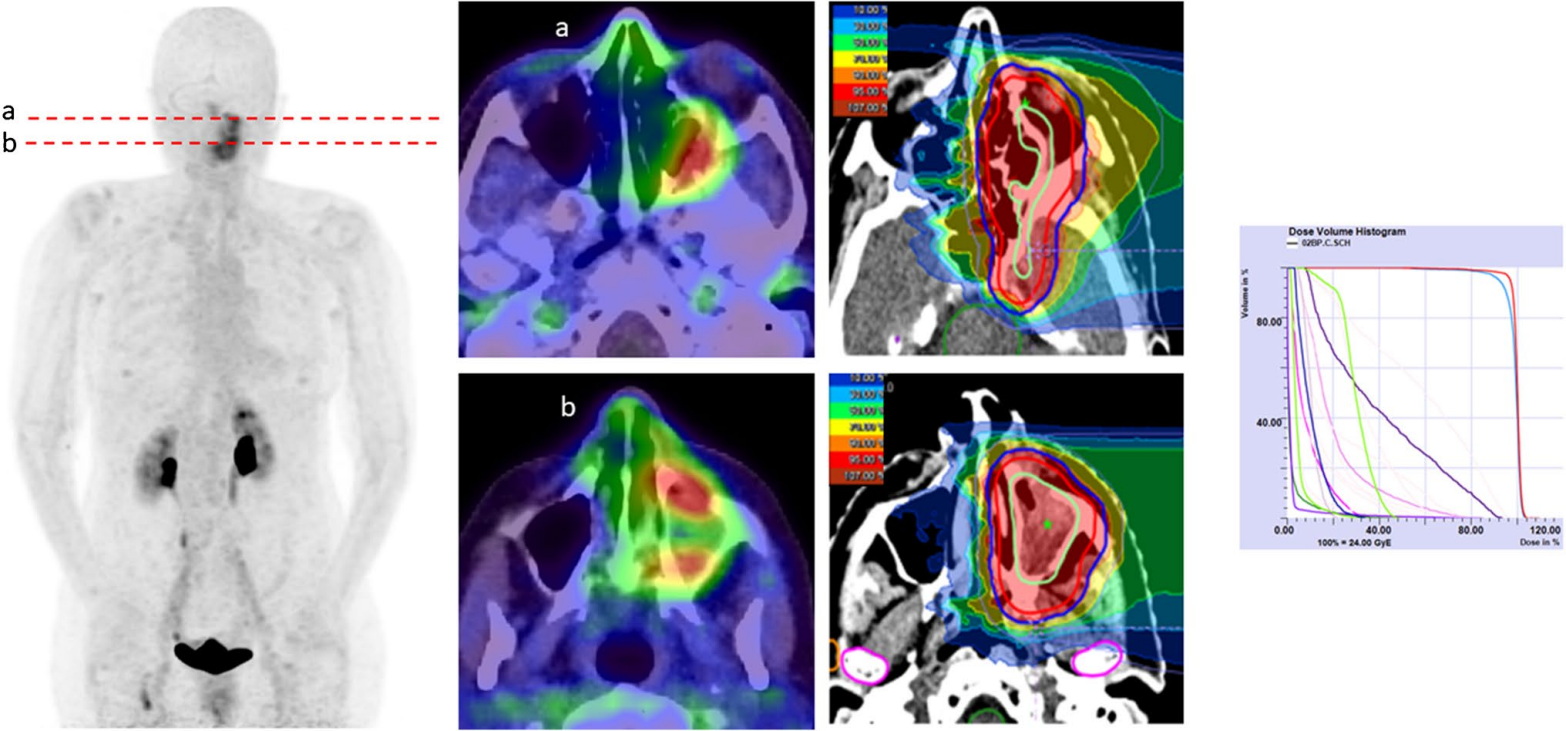
A 71-year-old female patient with HPV-associated adenoid cystic carcinoma (SUVmax 6.7) located in the left fossa pterygopalatine underwent ^68^ Ga-FAPI-PET/CT for radiotherapy planning

One patient diagnosed with esthesioneuroblastoma, located retro-maxillary, even achieved higher uptake 10 min after tracer application than 1 h later (Fig. 5), representing a prerequisite of the capabilities of FAPI-PET/CT in head and neck cancers. This subgroup has proven strong uptakes, which can be interpreted with respect to its mostly epithelial origin, as 90% of all epithelial tumors have shown high FAP expression [7]. This is consistent with our analyses where all head and neck cancers with epithelial origin (*n* = 15) showed a notably higher mean SUVmax (10.11) than those of non-epithelial or unknown origin (*n* = 9) obtaining a SUVmax of 7.5. Head and neck cancers are known to often grow diffusely, making the discrimination between healthy and tumorous tissue difficult. Thus, FAPI-PET/CT might be of help producing sharp imaging contrasts with high tumor uptake and low background activity, represented by high TBRs. This may also facilitate therapy guidance as the main approach is radiotherapy or combined radiochemotherapy, which requires precise and accurate tumor delineation (Fig. 6) [27].

Moreover, theranostic application of FAP ligands may play a fundamental role in some of these tumor entities in the future. A first case report by Kratochwil et al. described Samarium-labeled FAPI radioligand therapy in a patient with sarcoma achieving stable disease [28]. This case underlines the evidence of the exciting translation research from FAP imaging to FAP therapy.

**Fig. 7:**
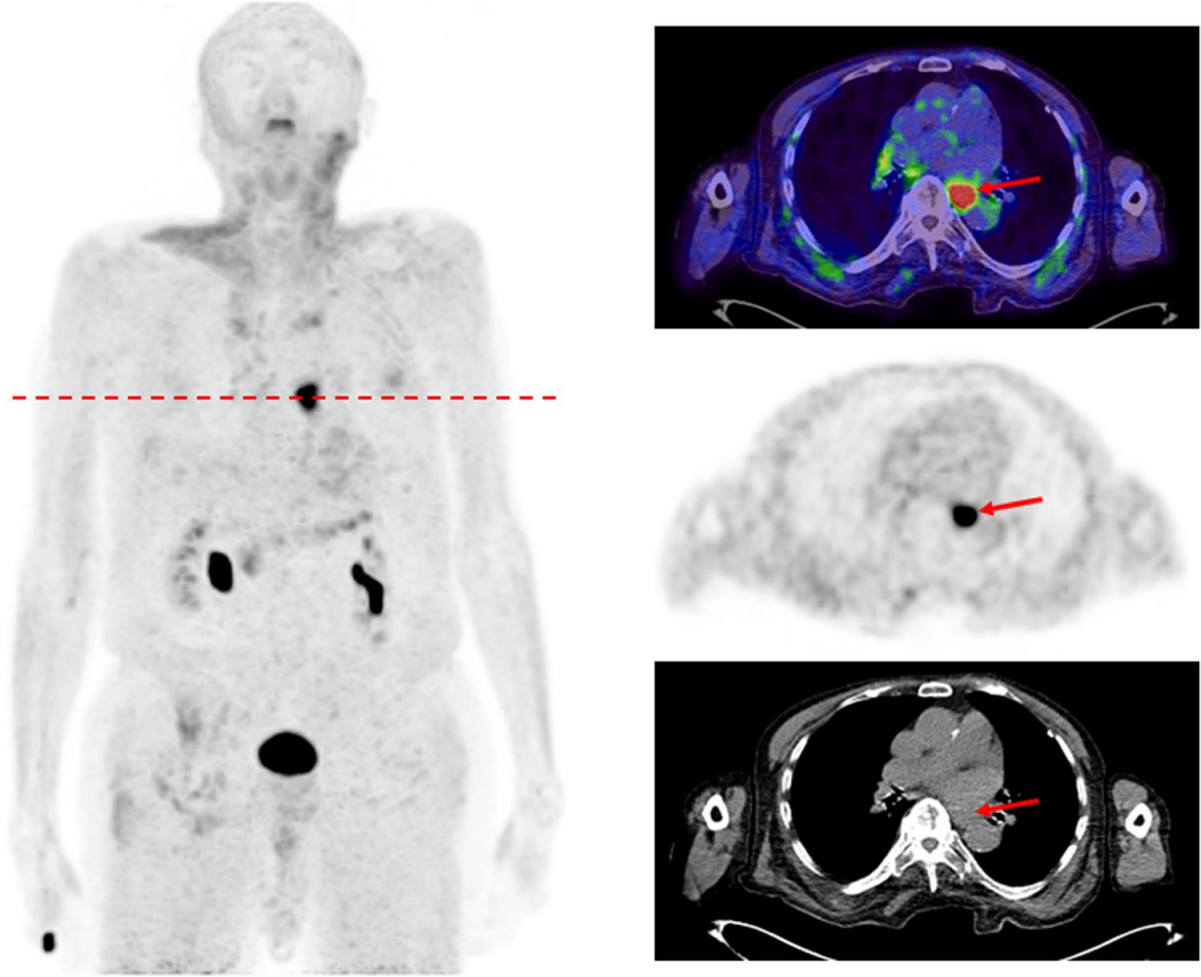
An 88-year-old male patient was diagnosed with a cancer of unknown primary and therefore underwent ^68^ Ga-FAPI-PET/CT for identification of the primary tumor localization. Remarkably, the FAPI scan showed an intense accumulation in the esophagus with a SUVmax of 16.29. This finding was subsequently histologically confirmed as a squamous cell carcinoma of the esophagus and treated accordingly

To find the most suitable therapeutic approach, knowing the origin of the primary tumor is of inevitable necessity. Lack of knowledge regarding tissue origin leads to a substantially more challenging treatment plan. FAPI-PET/CT was able to determine the location of the primary tumor and subsequently guide histologically confirmation, in a patient, where standard diagnostic imaging was insufficient beforehand (Fig. 7).

This retrospective single-center analysis contained several limitations. We used three different FAP ligands, yet biodistribution showed no significant differences regarding mean SUVmax (FAPI-46: 1.3 vs. FAPI 74: 1.4 vs. FAPI-04: 1.3). Within the scope of diagnostic evaluation, tumor uptake seems to be comparable according to previously conducted studies [12, 13]. The included subgroups differed in the number of patients limiting comparative analysis. No comparison with the current standard oncological tracer ^18^FDG was conducted. Furthermore, some diseases, even if declared rare by definition, occur fairly frequent such as HCC or urothelial carcinoma. Additionally, a variety of definitions of rare diseases are existing worldwide. However, a prevalence of no more than 1 person out of 2000 seems to be in concordance with the global average prevalence threshold [29]. Lacking a gold standard validation of discrepant lesion evaluation of sensitivity, specificity, and accuracy was beyond the scope of this work.

## Conclusion

^68^ Ga-FAPI-PET/CT crystalizes as a highly valuable and powerful molecular imaging technique in several rare tumor entities due to high and rapid tracer uptake in primary and secondary lesions, as well as low background activity, leading to sharp contrasts represented by excellent TBRs. On this basis, it may emerge in the future as an enrichment to standard diagnostic imaging and invites novel therapeutic interventions with respect to FAP phenotyping and FAP derivatives for early response therapy evaluation. Therefore, it possibly represents an indispensable imaging methodology in evaluating various malignancies, particularly epithelial carcinomas. Further research with increased patient numbers and statistical reliability is of essence to assess the immense potential of FAPI-PET/CT.

## Supplementary Information

Below is the link to the electronic supplementary material.

Supplementary file1 (DOCX 23 KB)
