# Supplementary material for: FAP imaging in rare cancer entities—first clinical experience in a broad spectrum of malignancies
Source: Eur J Nucl Med Mol Imaging. 2021 Aug 3;49(2):721–31. doi: 10.1007/s00259-021-05488-9 (PMC8803688; doi:10.1007/s00259-021-05488-9)
Supplement: Supplementary file 1 — Supplementary file1 (DOCX 23 KB) [file 259_2021_5488_MOESM1_ESM.docx]

| **Patient no.** | **Diagnosis** | **Cancer Status** | **Sex** | **Age (y)** | **Tracer** | **MBq** |
| --- | --- | --- | --- | --- | --- | --- |
| 1 | Cancer of unknown primary | 3 | m | 62 | ^68^Ga-FAPI-74 | 143 |
| 2 | Cancer of unknown primary | 3 | m | 51 | ^68^Ga-FAPI-74 | 265 |
| 3 | Cancer of unknown primary | 3 | w | 36 | ^68^Ga-FAPI-74 | 255 |
| 4 | Cancer of unknown primary | 3 | w | 59 | ^68^Ga-FAPI-4 | 274 |
| 5 | Cancer of unknown primary | 3 | m | 45 | ^68^Ga-FAPI-4 | 251 |
| 6 | Cancer of unknown primary | 3 | w | 76 | ^68^Ga-FAPI-4 | 245 |
| 7 | Cancer of unknown primary | 3 | m | 72 | ^68^Ga-FAPI-4 | 245 |
| 8 | Cancer of unknown primary | 3 | m | 78 | ^68^Ga-FAPI-46 | 216 |
| 9 | Cancer of unknown primary | 3 | m | 64 | ^68^Ga-FAPI-4 | 285 |
| 10 | Cancer of unknown primary | 1,3 | m | 88 | ^68^Ga-FAPI-4 | 183 |
| 11 | HPV induced Adenoid cystic carcinoma | 1 | w | 71 | ^68^Ga-FAPI-74 | 269 |
| 12 | Mucoepidermoid carcinoma | 1 | m | 24 | ^68^Ga-FAPI-74 | 241 |
| 13 | Parotid cancer | 1 | m | 59 | ^68^Ga-FAPI-4 | 278 |
| 14 | Parotid cancer | 1 | w | 77 | ^68^Ga-FAPI-4 | 164 |
| 15 | Parotid cancer | 1 | m | 80 | ^68^Ga-FAPI-4 | 157 |
| 16 | Parotid cancer | 1 | m | 31 | ^68^Ga-FAPI-46 | 277 |
| 17 | Tongue base cancer | 1,3 | m | 53 | ^68^Ga-FAPI-4 | 198 |
| 18 | Tongue base cancer | 3 | m | 76 | ^68^Ga-FAPI-4 | 241 |
| 19 | Tongue base cancer | 3 | m | 79 | ^68^Ga-FAPI-46 | 259 |
| 20 | Oral cavity cancer | 1 | m | 56 | ^68^Ga-FAPI-46 | 240 |
| 21 | Squamous cell carcinoma nose | 2 | m | 74 | ^68^Ga-FAPI-46 | 266 |
| 22 | Pituitary carcinoma | 1,3 | m | 48 | ^68^Ga-FAPI-46 | 266 |
| 23 | Esthesioneuroblastoma | 2,3 | m | 77 | ^68^Ga-FAPI-46 | 252 |
| 24 | Papillary cancer | 1 | m | 73 | ^68^Ga-FAPI-74 | 285 |
| 25 | Appendiceal cancer | 3 | m | 59 | ^68^Ga-FAPI-74 | 261 |
| 26 | Appendiceal cancer | 1 | w | 57 | ^68^Ga-FAPI-74 | 274 |
| 27 | Gastric cancer | 1,3 | w | 70 | ^68^Ga-FAPI-74 | 287 |
| 28 | Gastric cancer | 1 | w | 34 | ^68^Ga-FAPI-4 | 254 |
| 29 | Gastric cancer | 2 | m | 61 | ^68^Ga-FAPI-46 | 245 |
| 30 | Gastric cancer | 3 | w | 60 | ^68^Ga-FAPI-4 | 252 |
| 31 | Gastric cancer | 3 | m | 44 | ^68^Ga-FAPI-46 | 196 |
| 32 | Gastric cancer | 3 | w | 16 | ^68^Ga-FAPI-46 | 246 |
| 33 | Cecum cancer | 1 | m | 71 | ^68^Ga-FAPI-74 | 288 |
| 34 | Hepatocellular carcinoma | 1 | w | 85 | ^68^Ga-FAPI-4 | 237 |
| 35 | Hepatocellular carcinoma | 1 | m | 70 | ^68^Ga-FAPI-46 | 257 |
| 36 | Cholangiocarcinoma | 1,3 | m | 67 | ^68^Ga-FAPI-4 | 182 |
| 37 | Cholangiocarcinoma | 2,3 | m | 56 | ^68^Ga-FAPI-4 | 242 |
| 38 | Cholangiocarcinoma | 1,3 | m | 82 | ^68^Ga-FAPI-4 | 270 |
| 39 | Cholangiocarcinoma | 3 | m | 60 | ^68^Ga-FAPI-46 | 253 |
| 40 | Cholangiocarcinoma | 2,3 | m | 56 | ^68^Ga-FAPI-46 | 252 |
| 41 | Urothelial carcinoma | 1 | m | 62 | ^68^Ga-FAPI-46 | 216 |
| 42 | Urothelial carcinoma | 3 | m | 68 | ^68^Ga-FAPI-46 | 258 |
| 43 | Urothelial carcinoma | 1,3 | m | 75 | ^68^Ga-FAPI-74 | 141 |
| 44 | Urothelial carcinoma | 3 | m | 79 | ^68^Ga-FAPI-4 | 340 |
| 45 | Neuroendocrine tumor | 2,3 | m | 55 | ^68^Ga-FAPI-46 | 236 |
| 46 | Neuroendocrine tumor | 3 | m | 50 | ^68^Ga-FAPI-46 | 258 |
| 47 | Neuroendocrine tumor | 3 | m | 54 | ^68^Ga-FAPI-4 |  |
| 48 | Neuroendocrine tumor | 1 | w | 57 | ^68^Ga-FAPI-4 | 118 |
| 49 | Epitheloid hemangioendothelioma | 1,3 | w | 51 | ^68^Ga-FAPI-74 | 220 |
| 50 | Cutaneous squamous cell carcinoma | 1 | m | 85 | ^68^Ga-FAPI-4 | 279 |
| 51 | Malignant melanoma | 1 | m | 62 | ^68^Ga-FAPI-46 | 201 |
| 52 | Glomus tumor | 2,3 | w | 20 | ^68^Ga-FAPI-46 | 241 |
| 53 | Multiple myeloma | 1 | m | 59 | ^68^Ga-FAPI-74 | 239 |
| 54 | Follicular lymphoma | 1 | m | 40 | ^68^Ga-FAPI-46 | 259 |
| 55 | Tumor induced osteomalacia | 1 | m | 46 | ^68^Ga-FAPI-4 | 247 |

1 = primary tumor 2 = local relapse 3 = metastasized
